# Bis(μ-5-nitro-2-oxidobenzoato)bis­[triaqua­zinc(II)]

**DOI:** 10.1107/S1600536809045607

**Published:** 2009-11-04

**Authors:** Qing-Shan Li, Ma Yin, Hong Wei, Guang-Ju Zhou

**Affiliations:** a Key Laboratory of Metastable Materials Science and Technology, College of Materials Science and Engineering, Yanshan University, Qinhuangdao, Hebei Province 066004, People’s Republic of China

## Abstract

The title complex mol­ecule, [Zn_2_(C_7_H_3_NO_5_)_2_(H_2_O)_6_], is a centrosymmetric dimer containing two zinc(II) cations with distorted octa­hedral geometries provided by the O atoms of three water mol­ecules and the two bridging bidentate 5-nitro­salicylate ligands. The separation between the metal centres in the dimer is 3.1790 (11) Å. The crystal structure is stabilized by O—H⋯O hydrogen bonds, one of which intra­dimeric, linking the dimers into a three-dimensional network.

## Related literature

For examples of bonding modes exhibited by salicylate anions, see: Klug *et al.* (1958 ▶); Risannen *et al.* (1987 ▶); Charles *et al.* (1983 ▶); Jagner *et al.* (1976 ▶); Fu *et al.* (2005 ▶). For the crystal structures of 5-nitro­salicylate zinc(II) complexes, see: Tahir *et al.* (1997 ▶); Morgant *et al.* (2006 ▶); Erxleben (2001 ▶).
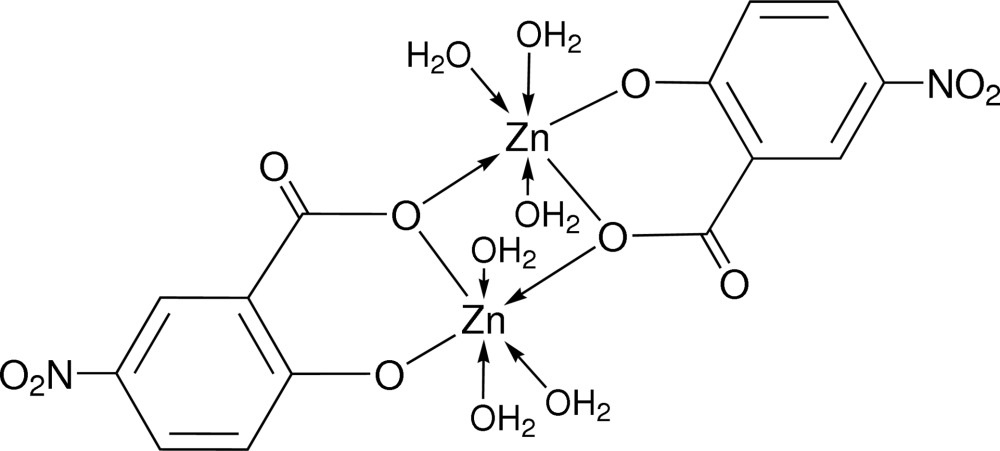


## Experimental

### 

#### Crystal data

[Zn_2_(C_7_H_3_NO_5_)_2_(H_2_O)_6_]
*M* *_r_* = 601.04
Monoclinic, 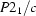

*a* = 10.858 (3) Å
*b* = 13.645 (3) Å
*c* = 6.6367 (17) Å
β = 91.887 (4)°
*V* = 982.7 (4) Å^3^
*Z* = 2
Mo *K*α radiation
μ = 2.53 mm^−1^
*T* = 294 K
0.26 × 0.10 × 0.08 mm

#### Data collection

Bruker SMART CCD area-detector diffractometer
Absorption correction: multi-scan (*SADABS*; Sheldrick, 2000 ▶) *T*_min_ = 0.752, *T*_max_ = 0.821
5435 measured reflections
2009 independent reflections
1418 reflections with *I* > 2σ(*I*)
*R*_int_ = 0.041

#### Refinement

*R*[*F* ^2^ > 2σ(*F* ^2^)] = 0.036
*wR*(*F* ^2^) = 0.088
*S* = 1.03
2009 reflections
154 parameters
H-atom parameters constrained
Δρ_max_ = 0.45 e Å^−3^
Δρ_min_ = −0.55 e Å^−3^

### 

Data collection: *SMART* (Bruker, 1997 ▶); cell refinement: *SAINT* (Bruker, 1997 ▶); data reduction: *SAINT*; program(s) used to solve structure: *SHELXS97* (Sheldrick, 2008 ▶); program(s) used to refine structure: *SHELXL97* (Sheldrick, 2008 ▶); molecular graphics: *SHELXTL* (Sheldrick, 2008 ▶); software used to prepare material for publication: *SHELXTL*.

## Supplementary Material

Crystal structure: contains datablocks I, global. DOI: 10.1107/S1600536809045607/rz2375sup1.cif

Structure factors: contains datablocks I. DOI: 10.1107/S1600536809045607/rz2375Isup2.hkl

Additional supplementary materials:  crystallographic information; 3D view; checkCIF report

## Figures and Tables

**Table 1:** Hydrogen-bond geometry (Å, °)

*D*—H⋯*A*	*D*—H	H⋯*A*	*D*⋯*A*	*D*—H⋯*A*
O8—H8*B*⋯O3^i^	0.84	1.81	2.651 (3)	173
O8—H8*A*⋯O5^ii^	0.85	2.26	3.038 (4)	153
O7—H7*A*⋯O8^iii^	0.85	2.58	3.023 (4)	114
O7—H7*B*⋯O1^iv^	0.85	1.77	2.596 (4)	163
O6—H6*B*⋯O4^v^	0.85	1.87	2.696 (4)	164
O6—H6*A*⋯O8^vi^	0.85	2.30	3.135 (5)	168

Symmetry codes: (i) 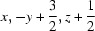; (ii) 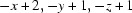; (iii) 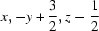; (iv) 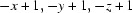; (v) 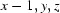; (vi) 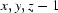.

**Fig. 1.**
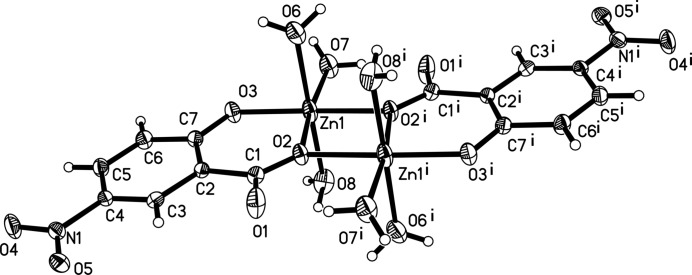
The structure of the title compound, with the atom-numbering scheme, and 30% probability displacement ellipsoids. H atoms are shown as small spheres of arbitrary radii and hydrogen bonds are indicated by dashed lines. [Symmetry code: (i) -x + 1, -y + 1, -z + 1.]

## supplementary crystallographic information

### Comment

From a coordination standpoint, salicylate is a versatile ligand displaying a variety of bonding modes. For example, mono-deprotonation of salicylic acid normally leads to complexes containing the coordinated 2-HOC_6_H_4_CO_2_ (salH) anion. This anion is known to bond to metals as a unidentate carboxylate *e.g.* in [Zn(salH)_2_(H_2_O)_2_] (Klug *et al.* 1958; Risannen *et al.*, 1987), as a bidentate chelating carboxylate *e.g.* in [Cd_2_(salH)_4_(H_2_O)_4_] (Charles *et al.* 1983), as a bidentate chelating ligand using one carboxylate oxygen and the hydroxyl oxygen *e.g.* in [Cu(salH)_2_].2H_2_O (Jagner *et al.*, 1976). On the other hand, deprotonation of both the hydroxyl and carboxyl protons from the parent acid generates the [OC_6_H_4_CO_2_]^2- ^(sal^2-^) anion, which can be found chelating through the phenolate oxygen and one of the carboxyl O atoms as in [Ti(sal)_3_]^2-^ (Fu, *et al.*, 2005).

Although many complexes which use salicylate as ligand have been synthesized, two structures coming out from the reaction of the 5-nitrosalicylic acid with zinc salt are known to us: a tetrahydrate (Tahir *et al.*, 1997), in an approximately octahedral geometry around the metal surrounding O atoms from four water ligands and two unidentate monoanionic 5-nitrosalicylate ligands using one carboxylate oxygen and a pentahydrate (Morgant, *et al.*, 2006), penta-aqua-(5-nitrosalicylato-O)-zinc(ii) 5-nitrosalicylate monohydrate, in which the metal is coordinated by five water ligands and one carboxylato O-atom from the 5-nitrosalicylato ligand. Interestingly, the title binuclear complex presents a third, different structure, being an hexahydrate dimer with its two zinc(II) atoms bridged by two carboxylate O atoms.

The structure of the title compounds is shown in Fig. 1. The distorted octahedral environment of each zinc(II) cation is defined by three O atoms from three water molecules, another two (the phenolate and a carboxylate one) from a chelating 5-nitrosalicylate and the centrosymmetric image of the latter. These two carboxylate O atoms bridge neighbouring zinc cations into a planar, four-membered matallacycle resulting in a Zn1···Zn1^i^ (see Fig 1 for symmetry codes) separation of 3.1790 (11) Å. It is the shortest of separation of Zn···Zn as reported previously in binuclear and tetranuclear zinc complex with salicylate ligands (Erxleben, 2001) It is worth mentioning that the use of a carboxylate oxygen as a bridging atom in salicylate metal complexes is rare; the title binuclear complex appears to be the first example of this behaviour in zinc complexes.

The carboxy group C1/O1/O2 as well as the nitro group N1/O4/O5 are effectively coplanar to the aromatic ring in the ligand as well as to the central Zn1/O2/C1/C2/C7/O3 six-membered ring generated upon coordination. The centrosymmetric character of the binuclear unit results in a large planar group composed of the two almost planar chelating ligands, the two zinc atoms and two O atoms from two aqua; the O atoms atoms from the remaining four aqua present Zn—O bonds almost orthogonal to this plane.

There are a number O—H···O hydrogen bonds stabilizing the structure (Table 1). The interaction involving O7—H7B and O1^i^ is intradimeric and coplanar to the dimer mean plane. The remainig ones define a three-dimensional framework.

### Experimental

The title complex was prepared by digesting a mixture of 5-nitrosalicylic acid (5 mmol) and fresh zinc hydroxide (10 mmol) in distilled water (30 ml) at 80 °C under stirring for 10 min. After filtration yellow-green crystals grew out of the solution by slow evaporation over a period of three days at room temperature. The starting zinc hydroxide was prepared from 50 ml aqueous solutions of 0.9 g of zinc chloride and 0.5 g of sodium hydroxide.

### Refinement

The H atoms of the water molecule were found in a difference Fourier map. However, during refinement, they were restrained to O–H = 0.85 (1) Å and their *U*_iso_ values were set at 1.2 *U*_eq_(O). Other H atoms were treated as riding, with C–H = 0.93 Å, and with *U*_iso_ (H) = 1.2 *U*_eq_(C).

### Crystal data

**Table:**

[Zn_2_(C_7_H_3_NO_5_)_2_(H_2_O)_6_]	*F*(000) = 608
*M**_r_* = 601.04	*D*_x_ = 2.031 Mg m^−^^3^
Monoclinic, *P*2_1_/*c*	Mo *K*α radiation, λ = 0.71073 Å
Hall symbol: -P 2ybc	Cell parameters from 1942 reflections
*a* = 10.858 (3) Å	θ = 2.4–25.8°
*b* = 13.645 (3) Å	µ = 2.53 mm^−^^1^
*c* = 6.6367 (17) Å	*T* = 294 K
β = 91.887 (4)°	Block, yellow-green
*V* = 982.7 (4) Å^3^	0.26 × 0.10 × 0.08 mm
*Z* = 2	

### Data collection

**Table:**

Bruker SMART CCD area-detector diffractometer	2009 independent reflections
Radiation source: fine-focus sealed tube	1418 reflections with *I* > 2σ(*I*)
graphite	*R*_int_ = 0.041
φ and ω scans	θ_max_ = 26.4°, θ_min_ = 1.9°
Absorption correction: multi-scan (*SADABS*; Sheldrick, 2000)	*h* = −13→13
*T*_min_ = 0.752, *T*_max_ = 0.821	*k* = −17→12
5435 measured reflections	*l* = −8→8

### Refinement

**Table:**

Refinement on *F*^2^	Primary atom site location: structure-invariant direct methods
Least-squares matrix: full	Secondary atom site location: difference Fourier map
*R*[*F*^2^ > 2σ(*F*^2^)] = 0.036	Hydrogen site location: inferred from neighbouring sites
*wR*(*F*^2^) = 0.088	H-atom parameters constrained
*S* = 1.02	*w* = 1/[σ^2^(*F*_o_^2^) + (0.0314*P*)^2^ + 1.0942*P*] where *P* = (*F*_o_^2^ + 2*F*_c_^2^)/3
2009 reflections	(Δ/σ)_max_ = 0.001
154 parameters	Δρ_max_ = 0.45 e Å^−^^3^
0 restraints	Δρ_min_ = −0.55 e Å^−^^3^

### Special details

**Table:**

Geometry. All e.s.d.'s (except the e.s.d. in the dihedral angle between two l.s. planes) are estimated using the full covariance matrix. The cell e.s.d.'s are taken into account individually in the estimation of e.s.d.'s in distances, angles and torsion angles; correlations between e.s.d.'s in cell parameters are only used when they are defined by crystal symmetry. An approximate (isotropic) treatment of cell e.s.d.'s is used for estimating e.s.d.'s involving l.s. planes.
Refinement. Refinement of *F*^2^ against ALL reflections. The weighted *R*-factor *wR* and goodness of fit *S* are based on *F*^2^, conventional *R*-factors *R* are based on *F*, with *F* set to zero for negative *F*^2^. The threshold expression of *F*^2^ > σ(*F*^2^) is used only for calculating *R*-factors(gt) *etc*. and is not relevant to the choice of reflections for refinement. *R*-factors based on *F*^2^ are statistically about twice as large as those based on *F*, and *R*- factors based on ALL data will be even larger.

### Fractional atomic coordinates and isotropic or equivalent isotropic displacement parameters (Å^2^)

**Table:**

	*x*	*y*	*z*	*U*_iso_*/*U*_eq_	
Zn1	0.52129 (4)	0.61159 (3)	0.44189 (8)	0.03665 (16)	
O1	0.7644 (3)	0.37686 (19)	0.3938 (5)	0.0555 (9)	
O2	0.6145 (2)	0.48166 (16)	0.4439 (4)	0.0310 (6)	
O3	0.6603 (2)	0.67583 (17)	0.3118 (4)	0.0364 (6)	
O4	1.2058 (2)	0.6122 (2)	0.1104 (5)	0.0506 (8)	
O5	1.1653 (2)	0.4656 (2)	0.2105 (4)	0.0393 (7)	
O6	0.4498 (3)	0.5753 (2)	0.1270 (5)	0.0564 (8)	
H6A	0.4933	0.5848	0.0249	0.068*	
H6B	0.3721	0.5766	0.1097	0.068*	
O7	0.4029 (3)	0.72837 (19)	0.4384 (5)	0.0507 (8)	
H7B	0.3400	0.7042	0.4931	0.061*	
H7A	0.3907	0.7724	0.3504	0.061*	
O8	0.5896 (3)	0.64083 (19)	0.7437 (4)	0.0493 (8)	
H8A	0.6412	0.5961	0.7716	0.059*	
H8B	0.6131	0.6997	0.7546	0.059*	
N1	1.1340 (3)	0.5505 (2)	0.1763 (5)	0.0333 (7)	
C1	0.7233 (3)	0.4611 (2)	0.3858 (5)	0.0271 (8)	
C2	0.8053 (3)	0.5409 (2)	0.3128 (5)	0.0242 (7)	
C3	0.9258 (3)	0.5143 (3)	0.2764 (5)	0.0273 (8)	
H3	0.9503	0.4496	0.2969	0.033*	
C4	1.0097 (3)	0.5813 (3)	0.2108 (5)	0.0281 (8)	
C5	0.9774 (3)	0.6787 (3)	0.1786 (6)	0.0333 (9)	
H5A	1.0350	0.7236	0.1346	0.040*	
C6	0.8604 (3)	0.7068 (3)	0.2126 (6)	0.0336 (9)	
H6	0.8387	0.7719	0.1905	0.040*	
C7	0.7692 (3)	0.6412 (2)	0.2803 (5)	0.0268 (8)	

### Atomic displacement parameters (Å^2^)

**Table:**

	*U*^11^	*U*^22^	*U*^33^	*U*^12^	*U*^13^	*U*^23^
Zn1	0.0288 (2)	0.0205 (2)	0.0615 (3)	−0.00197 (18)	0.01434 (19)	−0.0010 (2)
O1	0.0525 (18)	0.0247 (16)	0.092 (2)	0.0100 (12)	0.0359 (17)	0.0158 (15)
O2	0.0268 (13)	0.0184 (12)	0.0483 (17)	−0.0017 (10)	0.0091 (11)	−0.0007 (11)
O3	0.0268 (14)	0.0236 (13)	0.0593 (19)	0.0032 (10)	0.0096 (12)	0.0079 (12)
O4	0.0274 (14)	0.0560 (19)	0.069 (2)	−0.0043 (14)	0.0138 (13)	0.0088 (16)
O5	0.0314 (14)	0.0428 (17)	0.0436 (17)	0.0106 (12)	0.0012 (12)	0.0017 (13)
O6	0.0329 (16)	0.076 (2)	0.060 (2)	−0.0017 (15)	0.0045 (14)	−0.0002 (17)
O7	0.0429 (17)	0.0279 (16)	0.082 (2)	0.0012 (12)	0.0102 (16)	0.0103 (14)
O8	0.064 (2)	0.0238 (14)	0.061 (2)	−0.0075 (13)	0.0134 (15)	−0.0095 (13)
N1	0.0264 (16)	0.044 (2)	0.0293 (18)	−0.0002 (14)	0.0000 (13)	−0.0024 (14)
C1	0.0311 (19)	0.0213 (18)	0.029 (2)	0.0013 (14)	0.0042 (15)	−0.0004 (14)
C2	0.0243 (17)	0.0244 (18)	0.0240 (19)	−0.0004 (13)	0.0021 (14)	0.0008 (14)
C3	0.032 (2)	0.0263 (19)	0.023 (2)	0.0022 (14)	0.0015 (15)	0.0017 (15)
C4	0.0221 (17)	0.039 (2)	0.023 (2)	0.0008 (14)	0.0012 (14)	0.0003 (15)
C5	0.0287 (19)	0.034 (2)	0.037 (2)	−0.0037 (15)	0.0050 (16)	0.0027 (17)
C6	0.035 (2)	0.0226 (19)	0.044 (2)	0.0003 (15)	0.0051 (17)	0.0069 (16)
C7	0.0271 (18)	0.0243 (18)	0.029 (2)	0.0021 (14)	0.0036 (14)	0.0015 (14)

### Geometric parameters (Å, °)

**Table:**

Zn1—O3	1.969 (2)	O7—H7A	0.8457
Zn1—O2	2.041 (2)	O8—H8A	0.8452
Zn1—O7	2.047 (3)	O8—H8B	0.8448
Zn1—O2^i^	2.107 (2)	N1—C4	1.440 (4)
Zn1—O8	2.150 (3)	C1—C2	1.497 (5)
Zn1—O6	2.260 (3)	C2—C3	1.386 (5)
O1—C1	1.234 (4)	C2—C7	1.439 (5)
O2—C1	1.285 (4)	C3—C4	1.372 (5)
O3—C7	1.297 (4)	C3—H3	0.9300
O4—N1	1.237 (4)	C4—C5	1.389 (5)
O5—N1	1.226 (4)	C5—C6	1.353 (5)
O6—H6A	0.8486	C5—H5A	0.9300
O6—H6B	0.8486	C6—C7	1.418 (5)
O7—H7B	0.8511	C6—H6	0.9300
			
O3—Zn1—O2	90.14 (10)	Zn1—O8—H8B	110.5
O3—Zn1—O7	97.96 (11)	H8A—O8—H8B	118.1
O2—Zn1—O7	170.84 (10)	O5—N1—O4	122.3 (3)
O3—Zn1—O2^i^	169.08 (10)	O5—N1—C4	120.1 (3)
O2—Zn1—O2^i^	79.97 (10)	O4—N1—C4	117.5 (3)
O7—Zn1—O2^i^	91.59 (11)	O1—C1—O2	121.7 (3)
O3—Zn1—O8	94.59 (11)	O1—C1—C2	118.2 (3)
O2—Zn1—O8	89.95 (11)	O2—C1—C2	120.1 (3)
O7—Zn1—O8	93.63 (12)	C3—C2—C7	118.5 (3)
O2^i^—Zn1—O8	90.07 (10)	C3—C2—C1	116.2 (3)
O3—Zn1—O6	86.38 (11)	C7—C2—C1	125.4 (3)
O2—Zn1—O6	88.33 (11)	C4—C3—C2	121.4 (3)
O7—Zn1—O6	87.93 (12)	C4—C3—H3	119.3
O2^i^—Zn1—O6	88.69 (11)	C2—C3—H3	119.3
O8—Zn1—O6	178.03 (11)	C3—C4—C5	121.3 (3)
C1—O2—Zn1	130.4 (2)	C3—C4—N1	119.4 (3)
C1—O2—Zn1^i^	129.6 (2)	C5—C4—N1	119.2 (3)
Zn1—O2—Zn1^i^	100.03 (10)	C6—C5—C4	118.6 (3)
C7—O3—Zn1	128.7 (2)	C6—C5—H5A	120.7
Zn1—O6—H6A	121.5	C4—C5—H5A	120.7
Zn1—O6—H6B	115.5	C5—C6—C7	122.9 (3)
H6A—O6—H6B	117.7	C5—C6—H6	118.5
Zn1—O7—H7B	101.9	C7—C6—H6	118.5
Zn1—O7—H7A	130.2	O3—C7—C6	118.1 (3)
H7B—O7—H7A	117.4	O3—C7—C2	124.6 (3)
Zn1—O8—H8A	106.0	C6—C7—C2	117.3 (3)
			
O3—Zn1—O2—C1	2.4 (3)	O2—C1—C2—C7	−6.9 (5)
O2^i^—Zn1—O2—C1	177.8 (4)	C7—C2—C3—C4	0.0 (5)
O8—Zn1—O2—C1	−92.1 (3)	C1—C2—C3—C4	−179.4 (3)
O6—Zn1—O2—C1	88.8 (3)	C2—C3—C4—C5	0.1 (6)
O3—Zn1—O2—Zn1^i^	−175.34 (12)	C2—C3—C4—N1	179.6 (3)
O2^i^—Zn1—O2—Zn1^i^	0.0	O5—N1—C4—C3	−2.0 (5)
O8—Zn1—O2—Zn1^i^	90.08 (11)	O4—N1—C4—C3	177.5 (3)
O6—Zn1—O2—Zn1^i^	−88.96 (12)	O5—N1—C4—C5	177.5 (3)
O2—Zn1—O3—C7	−8.9 (3)	O4—N1—C4—C5	−3.0 (5)
O7—Zn1—O3—C7	175.4 (3)	C3—C4—C5—C6	−0.2 (6)
O2^i^—Zn1—O3—C7	−33.9 (7)	N1—C4—C5—C6	−179.7 (3)
O8—Zn1—O3—C7	81.0 (3)	C4—C5—C6—C7	0.2 (6)
O6—Zn1—O3—C7	−97.2 (3)	Zn1—O3—C7—C6	−170.1 (3)
Zn1—O2—C1—O1	−178.2 (3)	Zn1—O3—C7—C2	8.7 (5)
Zn1^i^—O2—C1—O1	−1.1 (5)	C5—C6—C7—O3	178.8 (4)
Zn1—O2—C1—C2	3.9 (5)	C5—C6—C7—C2	−0.1 (6)
Zn1^i^—O2—C1—C2	−178.9 (2)	C3—C2—C7—O3	−178.8 (3)
O1—C1—C2—C3	−5.5 (5)	C1—C2—C7—O3	0.5 (6)
O2—C1—C2—C3	172.4 (3)	C3—C2—C7—C6	0.0 (5)
O1—C1—C2—C7	175.2 (4)	C1—C2—C7—C6	179.3 (3)

Symmetry codes: (i) −*x*+1, −*y*+1, −*z*+1.

### Hydrogen-bond geometry (Å, °)

**Table:**

*D*—H···*A*	*D*—H	H···*A*	*D*···*A*	*D*—H···*A*
O8—H8B···O3^ii^	0.84	1.81	2.651 (3)	173
O8—H8A···O5^iii^	0.85	2.26	3.038 (4)	153
O7—H7A···O8^iv^	0.85	2.58	3.023 (4)	114
O7—H7B···O1^i^	0.85	1.77	2.596 (4)	163
O6—H6B···O4^v^	0.85	1.87	2.696 (4)	164
O6—H6A···O8^vi^	0.85	2.30	3.135 (5)	168

Symmetry codes: (ii) *x*, −*y*+3/2, *z*+1/2; (iii) −*x*+2, −*y*+1, −*z*+1; (iv) *x*, −*y*+3/2, *z*−1/2; (i) −*x*+1, −*y*+1, −*z*+1; (v) *x*−1, *y*, *z*; (vi) *x*, *y*, *z*−1.
